# Single-Cell RNA Sequencing Shows Markedly Elevated Immune Cell Proportions in the Endometrium of Women with Endometriosis—A Narrative Review

**DOI:** 10.3390/ijms27167440

**Published:** 2026-08-20

**Authors:** Muhammad Assad Riaz, Felix Zeppernick, Magdalena Zeppernick, Ivo Meinhold-Heerlein, Lutz Konrad

**Affiliations:** Center of Gynecology and Obstetrics, Faculty of Medicine, Justus Liebig University Giessen, 35392 Giessen, Germany; muhammad.a.riaz@gyn.med.uni-giessen.de (M.A.R.); felix.zeppernick@gyn.med.uni-giessen.de (F.Z.); magdalena.zeppernick@gyn.med.uni-giessen.de (M.Z.); ivo.meinhold-heerlein@gyn.med.uni-giessen.de (I.M.-H.)

**Keywords:** endometriosis, eutopic/ectopic endometrium, sc-RNA seq, pathogenesis, immune cells

## Abstract

Single-cell RNA sequencing is a state-of-the-art approach to decipher the heterogeneity of RNA transcripts in individual cells and identify different cell types in tissues and organs. Its aim is to create a high-resolution cell atlas to better understand organs and diseases. In this narrative review, we focus on the main endometrial cell types and their possible significance in endometriosis. Examination of the normal endometrium (NEM), eutopic endometrium of patients (EUE) and ectopic endometrium (EMS) revealed a predominance of endometrial stromal cells (ESCs), followed by epithelial cells (EECs), immune cells (ICs), endothelial cells (ECs) and pericytes (PVs). Comparison of the NEM with the EUE showed only minor changes in ESC and EEC proportions, but a dramatic increase in ICs in EUE, especially uterine macrophages (uM1). The ESC-IC homeostasis was especially disturbed. In EMS a marked reduction in EEC and NK cell proportions was found, accompanied by a significant increase in ECs and M2 macrophages. Furthermore, in EMS a significant shift from fibroblasts (Fibs) to myofibroblasts (MyoFibs), indicating fibrosis, was reported. Of note, ESC/EEC stem cells were described only very rarely, while nerve cells were not detected. In summary, scRNA-seq provided a good description of the cell proportions in NEM and EUE, but further studies are needed to analyze EMS. In conclusion, strong evidence supports the critical importance of ICs and immune system dysregulation in initiation and of ECs and MyoFibs in angiogenesis and fibrosis, respectively, in the pathogenesis of endometriosis.

## 1. Pathogenesis of Endometriosis

The basic features of endometriosis (EMS), such as pelvic pain (dysmenorrhea), infertility, diagnostic delay, etc., are generally known and have been summarized in many review articles, such as that by Bulun [1]. However, an important question that continues to be controversially discussed concerns pathogenesis [1]. Although Sampson’s implantation theory [2] is supported by most authors, several alternative explanations have been proposed, like the stem cell theory, the metaplasia theory, and the (epi-)genetic theory, to name but a few [1]. However, none of these alternative theories has yet been able to fully provide convincing explanations for the pathogenesis of EMS.

We are interested in the composition and role of endometrial cells in healthy endometrium (NEM), endometrium with EMS (EUE), and ectopic endometrium from endometriosis (EMS) lesions. The background is based on findings from a comparison of mRNA expression in NEM cells and EUE cells, where only very few differences of 0.92% were found [3]. Histologically, EUE cells also differ very little from NEM cells, in contrast to cancer cells [4], whose neoplastic precursors can be reliably identified in most cases. Therefore, the pathogenesis of EMS and cancer is only comparable to a very limited extent [3]. This observation also applies to ectopic cells in EMS lesions, which do not differ significantly from NEM and EUE cells histologically, so that the presence of ectopic endometrial epithelial and stromal cells is used by pathologists to diagnose EMS [4]. Furthermore, in a recently published review, we demonstrated that the mRNA expression of ectopic cells differs from that of eutopic cells by only 4.7% [3].

Since eutopic and ectopic endometrial cells are very similar both histologically and in terms of mRNA expression [3,4], we sought to determine whether the novel scRNA-seq technique could help to characterize the different endometrial cell types more accurately and reveal new differences. Single-cell RNA sequencing is currently the most advanced approach for deciphering the heterogeneity of RNA transcripts in individual cells and enables higher resolution of cellular differences [5]. This leads to a better understanding of the functions of individual cells in the context of their microenvironment [5]. The primary goal of scRNA-seq is to elucidate the composition of different cell types and their functions in tissues and organs in order to better understand organs and diseases [6].

## 2. Search Strategy, Inclusion, Exclusion Criteria, and Aims

This narrative review was conducted based on a literature search in PubMed covering the literature up to July 2025. The keywords endometriosis, normal, eutopic and ectopic endometrium in combination with scRNA-seq and RNA sequencing were used. Only manuscripts that analyzed at least normal endometrium, with or without eutopic and ectopic endometrium, were included (Figure S1). Pure reanalyses of published datasets were excluded, because they do not provide any information on cell numbers/proportions. The data from all manuscripts were extracted, including from the supplemental files, to evaluate and calculate the cell composition and cell numbers.

We aimed to summarize the identified scRNA-seq studies, with a particular focus on elucidating the composition of endometrial cells in NEM, EUE, and EMS. It was important for us to verify whether the scRNA-seq method yields a reliable high-resolution cellular atlas of the endometrium and endometriosis. Likewise, we sought to evaluate whether this approach facilitates a better understanding of the disease and this study is focused on the onset of endometriosis in the EUE. Assumptions regarding the mechanistic aspects of endometriosis based on scRNA-seq can be made reliably only if the cellular atlas is valid. Other mechanistic aspects of endometriosis than the immune system, fibrosis and angiogenesis as revealed by scRNA-seq have been covered in detail elsewhere [7,8].

## 3. Patients and Entities of Endometriosis Studied

In total, we found 14 publications examining native tissue (Table 1): EUE was compared with NEM in five studies, and among the EMS lesions, six publications focused on ovarian EMS (OMA), while only two studies investigated all three pelvic entities: OMA, peritoneal EMS (PE) and deep-infiltrating EMS (DIE) (Table 1). 

Fertility status was reported in most studies, but not in all [20,21]. This omission is notable, since infertility is an important problem in EMS [23]. The use of oral contraception (OC) was mentioned in four studies, whereas two studies did not report the use or non-use of OCs [11,12]. Unfortunately, none of the studies included reported on the removal of individual cells through cell picking, particularly from EMS lesions.

### 3.1. Cell Types in the Normal Endometrium

We have summarized all identified endometrial cell types from the various studies, demonstrating a very high degree of heterogeneity (Table 2). Three attempts were made to create a comprehensive endometrial cell atlas in order to reach a consensus [15,16,18].

The Human Endometrial Cell Atlas (HECA) was based on a combination of published [9,11,13,14,21,22] and newly generated data [15]. They classified the endometrial cells into: 1. ESCs (6 subclusters), 2. EECs (13 subclusters), 3. ECs (3 subclusters), and 4. ICs (2 subclusters). In addition, they also identified three perivascular/pericyte (PV) clusters and a C7-positive fibroblast (Fib) cluster.

Ulrich et al. [16] classified the NEM cell types into: 1. ESCs (11 subclusters), 2. EECs without cilia (8 subclusters), 3. EECs with cilia (1 subcluster), 4. blood ECs (5 subclusters), 5. PVs (3 subclusters) and 6. ICs (10 subclusters) and one lymphatic endothelial line [16].

Shin et al. [18] identified six major classes in NEM including: 1. ESCs + Fibs (10 subclusters), 2. EECs (9 subclusters), 3. blood ECs (7 subclusters), 4. PVs (4 subclusters), 5. lymphoid cells (14 subclusters), and 6. myeloid cells (14 subclusters).

Due to the very high heterogeneity in cell classifications, it can be summarized that these studies represent only the first steps toward the development of a comprehensive endometrial cell atlas based on sc-RNAseq.

### 3.2. Endometrial Stromal Cells

In the endometrial cell atlas generated by Ulrich et al. [16], 11 stromal subtypes (Stro-1–11) were characterized, including three PV lines (Stro-1–3), one tissue-resident vascular progenitor (Stro-4), one *RUNX3*+ Fib (Stro-5), one uterine SMC (Stro-6), one C7 Fib (Stro-7), three decidualized Fib lines (Stro-8–10, also labeled as dS1–3), and one proliferative progenitor cell type (Stro-11). However, Stro-1–4 belong to the PV and EC group. Although the authors have provided a comprehensive overview of all proliferative and secretory phases, we will only discuss the key differences between the proliferative (P) and late secretory (LS) phases (Table 3). The most pronounced decrease in cell numbers was observed in Stro-11 (22×) and Stro-5 (3×), in contrast to the increase in Stro-8 (8×) and Stro-6/7 (4×). In the LS phase, Stro-9 and Stro-10 are particularly prevalent, while Stro-8 displayed the strongest increase in the LS phase compared to the P phase. It is noteworthy that the decidualized cell lines Stro-9 and Stro-10 were present in comparable numbers during both the proliferative and late secretory phases, which is unexpected since decidualization is a characteristic feature of the secretory phase.

Marečková et al. [15] identified 15 mesenchymal cell types with three proliferative ESC lines (eStromal MMP11, eStromal, eStromal cycling), three secretory decidual ESC lines (dS-early, dS-mid, dS-late), one Fib Basalis (C7), three myometrial, two endocervical/cervical and two cell lines (hormones) derived from the use of contraception. We noticed that the Fib Basalis C7 resembles the Stro-7 [16]. Of note, the eStromal matrix metalloproteinase11 (*MMP11*) cells also showed upregulation of *MMP1*, *MMP10* and *MMP3*, and were especially evident during the menstrual and early proliferative phases [15], emphasizing the importance of MMPs for menstruation and tissue regeneration.

In the first scRNA-seq study [17], although 13 Fib subgroups were identified and striking similarities between ESCs in NEM and EUE were found, it was emphasized that four Fib subtypes (SC-FB1/6/8/9) were NEM-specific and four Fib subtypes (SC-FB2/3/10/11) were EMS-specific. The NEM-specific SC-FB-1/6/8/9 ESCs were more related to physiological functions such as gland development and tissue morphogenesis [17].

Shin et al. [18] identified five stromal cell types, including two Fib lines: Fib-1, a non-fibrotic line with C7 expression found in NEM and EMS lesions, and Fib-2 with pro-fibrotic features similar to the three MyoFib (labeled MFib1–3) lines.

In another study, two Fib and two MyoFib lines were classified, with the Fibs being the dominant cell type in the NEM alongside a MyoFib cluster [19]. In the proliferative NEM, Queckbörner et al. [10] reported an unusually high percentage of ESCs, which were further subdivided into eight populations. Of note, the ESC lines 1–3 demonstrated a profile typical for extracellular matrix (ECM) breakdown, remodeling and organization.

### 3.3. Endometrial Epithelial Cells

Ulrich et al. [16] identified nine endometrial EEC lines: 1. Epi-1 (ciliated), 2. Epi-2 (*MUC16*, glandular secretory), 3. Epi-3 (*SOX9*, glandular), 4. Epi-4 (luminal), 5. and 6. Epi-5 and Epi-6 (glandular secretory progenitor subtypes A and B, respectively), 7. Epi-7 (myoepithelial subtype resembling cells from the fallopian tube), 8. Epi-8 (*OLFM4*_proliferative) and 9. Epi-9 (tissue-resident memory T cells resembling cells from the fallopian tube). Overall, a significant EEC increase from the proliferative to the late secretory phase was observed, particularly for Epi-2, Epi-5, and Epi-6, while Epi-3 and Epi-8 occurred significantly less frequently in the LS phase (Table 4). Of note, the estrogen receptor-α (*ESR1*) was broadly expressed in all EEC subtypes, the progesterone receptor (*PGR*) was specifically high in Epi-3,7,8,9 and the androgen receptor (*AR*) was restricted to Epi-7.

In the HECA, 16 EEC lines were classified [15]: 1. four *SOX9*-expressing EECs (*SOX9* Basalis/*CDH2*, *SOX9* Functionalis I/*CDH2*, *SOX9* Functionalis II, *SOX9*/*LGR5* Luminal), 2. one cycling, 3. four glandular secretory (preglandular, glandular, secretory, glandular secretory/*FGF7*), 4. two luminal secretory (preluminal, luminal), 5. two ciliated (preciliated, ciliated), 6. two endocervical/cervical (*MUC5B*, *KRT5*), and 7. one line from patients using contraception.

Shin et al. [18] identified two luminal epithelial (LE) lines in addition to seven EECs, including ciliated, glandular, glandular secretory, *MUC5B*, *SOX9* prolif, *SOX9*/*LGR5*, and *SOX9*/*LGR5*. However, the *MUC5B* cells are most likely endocervical cell contamination, as suggested by Marečková et al. [15]. *MUC5B* is expressed almost exclusively in the endocervix, as shown by mRNA expression and immuno-histochemistry [23].

Luminal EECs were identified by many studies [9,11,14,15,16,18,21] but not by others [10,12,13,17,19,20,22]. In the HECA, three LE lineages are distinguished, a preluminal one expressing estrogen sulfotransferase 1 (*SULT1E1*) [15,21] and two luminal cell lines, one with expression of leucine-rich repeat containing G protein-coupled receptor 5 (*LGR5*) and SRY-box transcription factor 9 (*SOX9*) [11,15,18,21,22], although not consistently labeled as luminal by all authors, and a second one labeled as luminal secretory. In contrast, only one luminal cell line (Epi-4) was described in the other endometrial cell atlas [16]. Beyond the common expression of *LGR5*, these cells exhibit highly specific expression of *IL6* in the early secretory phase [16]. Similarly, luminal-1 and epithelial *SOX9*/*LGR5* cells also expressed *IL6* in a highly specific manner [18]. Only one study reported the expression of *LGR5* and *PTGS1* in all three LE cells [21].

### 3.4. Endometrial Endothelial and Perivascular Cells

Ulrich et al. [16] identified lymphatic ECs (LECs), eight blood EC subtypes (Endo-1 through Endo-8), and the tissue-resident vascular progenitor Stro-4 (Table 5). Interestingly, most of the eight EC subtypes corresponded to those described by Tan et al. [21]. The EndoMT (ECs undergoing mesenchymal transition), Tip, and high endothelial venule (HEV) abundances are clearly decreased, whereas tissue postcapillary venules (tPCVs) and activated PCVs (aPCVs) are increased in the late secretory phase compared to the proliferative phase. The EndoMT cells are the only EC subtype expressing both *ESR1* and *PGR* and are characterized by high expression of mesenchymal markers and reduced expression of classic EC markers [16]. Furthermore, three PV cell lines (Stro-1 to Stro-3) were identified, and only for Stro-1 a ~3-fold reduction in cell proportions in the late secretory phase compared to the proliferative phase was reported [16]. Stro-1 cells may be involved in the secretion of ECM molecules and thus likely in the stiffness or mobility of the matrix, while Stro-2 may be actively involved in angiogenesis. Ligand–receptor analysis suggested that Stro-3 cells may communicate with macrophages, NK cells, and CD8 cells [16]. The *STEAP4* metalloreductase-expressing Stro-3 cells are similar to ePV-1 [15] and PV-*STEAP4* [18].

In the HECA [15], three subsets of ECs (venous, arterial and lymphatic) and three subsets of PVs were identified in NEM and EUE (Table S1 and Table 2). The clear predominance of venous compared to arterial blood vessels was also shown by Ulrich et al. [16].

Shin et al. [18] classified seven EC subtypes similarly to the HECA [15] and the study by Ulrich et al. [16]. They found that the venous-2 ECs were the most abundant, followed by the capillary and arterial ECs. Furthermore, they identified four PV subtypes: 1. PV-Steap4, 2. PV-*MYH11*, 3. PV-diff., and 4. PV-*CCL19*. The PV-*CCL19* cells might promote angiogenesis and facilitate IC trafficking [21].

### 3.5. Immune Cells and the Immune System in Endometriosis

Ten IC subtypes, Imm-1 to Imm-10, were classified by Ulrich et al. [16] (Table 6). The Imm-2 fibrocytes were proposed to play an essential role in tissue repair, while the tissue-resident Imm-10 could facilitate a rapid response to local immune changes in the uterus. The most abundant IC types were the NK cells (Imm-9), the macrophages (Imm-8) and the T cells (Imm-3–5) (Table 6). The ICs were present throughout the menstrual cycle; notably, macrophage abundance increased 1.4-fold in the late secretory compared to the proliferative phase, suggesting a role in menstruation. In contrast, the proportion of innate lymphoid cells (ILCs) and TMEMCD8 cells dropped strongly from the proliferative to the late secretory phase.

In the HECA [15], the ICs were grouped into the immune lymphoid and immune myeloid lineages. The authors distinguished four main groups: 1. B cells (i1 = B cells; i2 = plasma B cells), 2. T cells (i3 = T cell CD4; i4 = T cell CD8; i4c = T cell cycling; i5 = T reg), 3. innate lymphocytes (i6 = uNK1; i6c = uNK1 cycling; i7 = uNK2; i8 = uNK3; i9 = ILC3; i10 = peripheral lymphocytes), and 4. the myeloid lineage (i11 = cDC1; i12 = cDC2; i13 = uM1; i14 = uM2; i14c = uM2 cycling; i15 = monocytes; i16 = mast cells; i17 = pDC; i18 = red blood cells). They used the following abbreviations: u = uterine; cDCs = conventional dendritic cells; and pDCs = plasmacytoid DCs. Marečková et al. [15] further analyzed the cell–cell communication between uterine macrophages (uMs) and NK cells (uNKs) with ECs and PVs. Altogether, the analysis suggested that uMs are the major endometrial ICs involved in blood vessel formation, wound healing and anti-inflammatory responses. The latter two processes are likely to aid the ESCs in healing without scarring [15]. However, these far-reaching hypotheses need to be substantiated by experimental evidence.

Ma et al. [17] published that T and NK cells were the most abundant IC types in NEM as well as in EUE, but B cells were not detected. Similarly to Marečková et al. [15], Ma et al. [17] also observed a broad communication between ICs and ESCs.

Shin et al. [18] compared the NEM ICs with all three pelvic entities (Table S2), and found the highest proportions for all T cells (50.2%), with particularly high values for T cytotoxic (Tc; 30.3%), followed by CD4Tem and cycling T (8.1% each). The total NK cell count was also very high in NEM (40.4%), with NK3 (19.5%) and NK2 (17.5%) standing out. Macrophages (eight cell populations) and dendritic cells (four cell populations) could not be included in this calculation because they were presented separately [18]. Similarly, Tan et al. [21] identified five macrophage subtypes.

Zhu et al. [19] compared NEM, EUE and EMS and classified the ICs into 1. macrophages (M1, M2, M proliferating), 2. T cells (CD8, CD4, naïve, proliferating), 3. NK cells, 4. mast cells, 5. plasmacytoid (p)DCs, 6. neutrophils, and 7. plasma B cells. In NEM the NK cells are the most abundant cell population, followed by T naïve, CD8 effector T cells and M2.

### 3.6. Comparison of NEM with EUE and EMS

We use the HECA as a basis, as most scRNA-seq studies were summarized in this study [15]. The percentages of the five major cell types in NEM and EUE were calculated and included four studies [11,14,15,21] that provided data for all five cell types (Table S3 and Table 7). The problems regarding the heterogeneity of cellular endometrial composition across various scRNA-seq studies have already been noted [15]. This heterogeneity may stem from hormonal treatment, the menstrual cycle, or differing cell isolation methods [15]. Nevertheless, the HECA was constructed using this heterogeneous data and stated the absence of an EMS-specific cell state [15]. Thus, we analyzed only data from samples without hormonal treatment and stratified them according to the proliferative and secretory phases. Furthermore, the *MUC5B*-positive cells were not included because these cells belong to the cervix [15]. We found the following ascending order in the proliferative NEM (Table S3 and Table 7): 1. ESCs 57.8%, 2. EECs 14.57%, 3. ICs 12.31%, 4. ECs 8.43% and 5. PVs 6.88%. We identified a clear decrease in ESCs of ~10%, in contrast to a clear increase in EECs of ~6.4% and in ICs of ~9.5% in EUE compared to NEM (Table 7). This resulted in an ESC/EEC ratio of ~4:1 for NEM and ~2.0:1 for EUE. This interesting observation, particularly during the proliferative phase, can be linked to the estrogen dominance known to be associated with endometriosis [1]. The effect diminishes during the secretory phase, where only a ~3.5% increase in IC count was observed in EUE compared to NEM (Table 7). However, these hypotheses, although highly likely, require further verification. The reduced proportion of ECs by ~3.0% in EUE vs. NEM in the proliferative phase was parallel to the 2.8% decrease in PVs. A similar trend was found in the secretory phase with a strong decrease of ~6.5% in ECs and of 1.1% in PVs in EUE compared to NEM. In the following section, we will discuss the changes from NEM to EUE and EMS for each major cell type.

The detailed ESC numbers were only specified for three proliferative and three secretory cell lines in the HECA [15]. The comparison of NEM with EUE shows a clear reduction in endometrial stromal cells specific to the proliferative phase (eStromal) and late secretory decidual stromal ESCs (dS-late) in contrast to a significant increase in early secretory decidual stromal (dS-early) and mid-secretory decidual stromal cells (dS-mid). Overall, significantly more decidual cells were found in EUE than in NEM (Table 8). For the EECs the proportions of cycling EECs and glandular (gland) EECs were increased, whereas the proportions of SOX9-positive EECs of the functionalis II (SOX9 funct II) and Glandular_secretory EECs were clearly decreased in EUE vs. NEM (Table 9).

In the three studies on NEM, EUE, and EMS [18,19,20], we summarized the ESC proportions despite all shortcomings (Table 10). Again, the proportion of ESCs was highest and showed only moderate differences in cell numbers but a strong shift in the ratio (~1:2) of Fibs to MyoFibs in EMS [19]. The three MyoFib (labeled MFib1–3) lines were found almost exclusively in the EMS lesions with distinct expression in the different PE subtypes [18]. Although the MyoFib lines shared several MyoFib marker genes, they also expressed subpopulation-specific genes. Similarly, the MyoFibs are much more abundant in EMS lesions compared to EUE and NEM and it was suggested that they were generated by a Fib to MyoFib transition (FMT). Of note, staining for KI-67 in the ESC lines was similar in EUE and NEM but much stronger compared to EMS lesions. Furthermore, the ESCs in the EMS lesions showed mostly ECM organization, as well as TGF-β and WNT signaling pathways, indicating increased fibrosis [19]. In another study, the NEM-specific ESCs (labeled SC-FB-1/6/8/9) were more related to physiological functions such as gland development and tissue morphogenesis [17], whereas the EMS-specific ESCs (labeled SC-FB-2/3/10/11) were enriched with functions including ECM structure organization, wound healing, and cell adhesion [17]. Of note, Shin et al. [18] showed significantly increased ESC proportions in OMA but markedly reduced percentages in DIE, suggesting two different pathogenetic pathways. However, the very high proportion of ESCs in OMA is surprising, as often only a very thin layer of ESCs is present around the ovarian cysts.

In another study that only examined EUE and OMA [22], four major ESC lines were identified: 1. membrane metalloendopeptidase (*MME*, CD10)-positive endometrial-type stroma (EnS; two subclusters), 2. Fibs (nine subclusters), 3. smooth muscle cells (SMCs; one cluster) and 4. bland cells expressing the growth arrest-specific 5 long noncoding RNA (*GAS5* cells; one cluster). The first two groups were further subdivided into proliferative ESCs (labeled proliferative EnS) and secretory ESCs (labeled secretory EnS). The comparison of EUE with OMA revealed that the distribution of proliferative and secretory ESCs did not align with the menstrual cycle phases, mirroring hormonal dysregulation [22]. Furthermore, in OMA ESCs, several genes, including ECM reorganization and multiple collagen genes, were upregulated, thereby supporting a pro-fibrotic phenotype.

In the three studies comparing all endometrial cell types in NEM, EUE and EMS [18,19,20], the percentages of EECs were reduced in EUE and particularly dramatically in EMS compared to NEM (Table 10). However, these results are not fully consistent with histological observations [4], as EECs are clearly identifiable in EMS lesions and their proportion is unlikely to be as low as 1.9% (Table 10). A preliminary study by us revealed that the EEC count drops from eutopic endometrium (EUE) to endometriotic lesions (EMS); however, the reduction is only ~35%. Based on these preliminary data for the EECs, we have corrected the mean values. There are only minor shifts in the proportions of the five major cell types, suggesting that the differences between NEM, EUE and EMS might be found within the subgroups. In one of these three studies [20], a significantly increased proportion of ciliated EECs was found in the EUE and especially in the EMS lesions compared to NEM. However, the data from Marečková et al. [15] do not confirm the increase in ciliated cells in the EUE. Only Tan et al. [21] identified three LE (LE1–3) cell lines and found significantly reduced percentages in EUE vs. NEM. Furthermore, the proportion of the LE1 lineage was particularly high in PE lesions, but the LE1 and LE2 cells were only slightly detectable in OMA lesions [21]. This study is the only scRNA-seq report to date in which the LE cell types were found in ectopic EMS lesions at all, an aspect that has received limited attention to date, especially since *PTGS2* (COX2) is preferentially expressed in LE EECs and some adjacent glands [24,25] and higher in ectopic glands [26].

It is very difficult to compare the IC proportions in NEM, EUE, and EMS because the data in the manuscripts have different reference values and the terminology also differs. Nevertheless, the comparison of NEM with EUE showed reduced total T cell counts, slightly increased values for all NK cells, but a strong, nearly 2-fold increase in the proportions of macrophages (Table 11). In the HECA [15], comparison of NEM with EUE demonstrated an increase from ~8.5% to ~20.7% of the lymphoid lineage, whereas only negligible changes in the myeloid lineage were observed (Table S4). Of note, Marečková et al. [15] detected a higher abundance of uM1 in EUE compared to NEM. Both uM1 and uM2 strongly expressed several MMPs and pro-angiogenic genes. An increased expression of (pro-)inflammatory genes in uM1 was found in EUE compared to NEM. In contrast, uM2 expressed anti-inflammatory and tissue-resident marker genes.

Notably, the EMS macrophages in particular expressed higher levels of adhesion molecules as well as higher levels of growth and angiogenesis factors, while the corresponding receptors are widely expressed in ESCs, which could enhance their adhesion and growth [15]. The EMS-specific SC-M-2 macrophages exhibited enriched functions including response to wounding and angiogenesis, while the SC-M-8 macrophages were related to ECM organization, wound healing, and cell adhesion. These results suggest that EMS-associated macrophages might be engaged in wound healing and tissue remodeling.

Similarly to Marečková et al. [15], Ma et al. [17] also observed a broad communication between ICs and ESCs. EMS ESCs compared to EUE ESCs produced higher levels of inflammatory factors (e.g., IL-6), while the corresponding receptors were expressed in mast cells and macrophages/monocytes. They also observed putative inhibitory interactions between ICs and ESCs in EMS rather than in EUE. Furthermore, they identified a decreased ratio of naïve and cytotoxic T cells in EMS vs. NEM and EUE, suggesting a hindrance in the clearance of disease lesions.

Shin et al. [18] compared the NEM ICs with all three pelvic entities (Table S2 and Table 3) and found the highest proportions for all T cells (50.2%), with particularly high values for T cytotoxic (Tcyt; 30.3%), followed by CD4 effector memory T cells (CD4Tem) and cycling T cells (8.1% each). In EMS, the total T cell count was significantly increased in all three entities examined, especially that of Tcyt, CD4Tem, and CD8Tem, while the percentages of cycling T cells decreased significantly. The total NK cell count was also very high in NEM (40.4%), with NK3 (19.5%) and NK2 (17.5%) standing out. In the three entities, the total numbers of NK cells were dramatically reduced, particularly affecting NK2 and NK3 cells. Additionally, they found that the lymphoid cells had high cytotoxic signatures in EMS; especially in DIE, the immunological and inflammatory signatures were altered severely compared to PE and OMA [18].

Only one study compared macrophage subtypes across NEM, EUE, and EMS [21]. A striking finding was that the proportion of infiltrating M4 macrophages decreased significantly from NEM to EUE and EMS (Table S5). A similar pattern was observed for M3 macrophages (*APOE*—apolipoprotein E-positive), which decreased by ~5% from NEM to EUE but were slightly higher again in peritoneal lesions. In contrast, the percentages of tissue-resident M1 macrophages (*FOLR2*—folate receptor beta-positive) rose dramatically—doubling from NEM to EUE and increasing 10-fold in OMA compared to NEM.

The summary of the EC percentages across four studies (Table S1) revealed a clear predominance of venous compared to arterial blood vessels, as also shown by others [16,18]. Furthermore, there was a clear decrease in venous and arterial ECs in EUE compared to NEM (Table S1). The lower percentages of ECs in EUE compared to NEM was also described in two other studies [18,20]. This is a very interesting observation and could indicate that the reduced number of ECs in EUE compared to NEM could contribute to EMS-associated infertility. To the best of our knowledge, only impaired remodeling of spiral arteries in the endometrium during pregnancy [27] and angiogenic dysfunction due to reduced *VEGFA* and angiogenesis-related protein expression [28] have been associated with infertility.

Shin et al. [18] reported that the venous-2 ECs were the most abundant EC subtype, followed by the capillary and arterial ECs (Table 12). Endothelial cells of venous-1, lymphatics (LECs), and tip cells (the leading cells of the sprouts) were abundant in EMS compared to NEM. In particular, venous-1 showed a marked increase in OMA, PE, and DIE compared to NEM, suggesting enhanced angiogenesis in EMS. In contrast, reduced percentages of venous-2 and cycling ECs in EMS compared to NEM were identified (Table 12). Overall, the ECs displayed pro-inflammation, angiogenesis, and leaky permeability signatures that were enhanced in EMS [18].

In the study by Zhu et al. [19], three EC subtypes (Endothelial.C1–C3) were identified. The proportion of Endothelial.C1 was significantly higher in EMS lesions than in EUE and NEM, while the number of Endothelial.C2 and Endothelial.C3 was negligible in EUE. It is noteworthy that the signaling pathways associated with vasculogenesis were mainly restricted to ECs and PVs, suggesting that both cell types play a key role in angiogenesis in EMS.

Three PV cell types (ePV1a, ePV1b, PV2) were classified in the HECA [15]. A remarkably high proportion of ePV2 was observed in both NEM and EUE, while ePV1a was almost completely absent in EUE. However, the authors did not elaborate on these aspects.

Shin et al. [18] identified four PV subtypes: PV-*STEAP4*, PV-*MYH11*, PV-diff, and PV-*CCL19*. All PV cell types combined had the lowest proportion in NEM, but an increased proportion in EUE and all three pelvic entities. Similarly, Tan et al. [21] claimed that PV-*CCL19* cells constitute the majority of PV cells in PE lesions and in the peritoneal periphery, but were not detected in OMA. Furthermore, they assumed a dual role for PV-*CCL19* cells in promoting angiogenesis and facilitating IC transport. Although Zhu et al. [19] identified only a single PV type, its proportion in EMS was significantly higher than in EUE and NEM. Furthermore, ECs showed the greatest differentiation potential compared to PVs, suggesting that PV differentiation may be EC-dependent.

### 3.7. Endometrial Stem Cells

In recent years, endometrial stem cells have increasingly become the focus of research and have been attributed important roles in re-epithelialization during menstruation, regeneration after menstruation and in the pathogenesis of EMS [29].

#### 3.7.1. Stromal Stem Cells

Stro-11 is a potential proliferative stromal precursor, expressing MKI67 and the cyclins *CCNB1/2* [16], suggesting a role in active tissue remodeling [16]. Interestingly, a similar expression pattern was found in the SC-FB8/9 lines, which were detected exclusively in NEM and EUE, but not in EMS lesions [17]. The authors hypothesized that uterine ESCs may undergo a mesenchymal–epithelial transition (MET) to contribute to the regeneration of the epithelial and endothelial compartments during the normal cycle [16]. However, recent sequencing data showed no transition in the endometrium, neither in the form of MET nor EMT [30]. Furthermore, *CCNB1/2* are present in EECs as well as ESCs [31], making the classification of Stro-11 as stromal precursors debatable.

#### 3.7.2. Epithelial Stem Cells

Epi-7 (myoepithelial) and Epi-8 (*OLFM4*_Prolif) have been suggested to be the epithelial progenitor cells for Epi-6 and then for other EECs [16], and these cells might be necessary for optimal tissue maintenance and regeneration. However, it was not proven that Epi-7 and Epi-8 are really stem cells. In the HECA a new population of SOX9/cadherin-2 (*SOX9*/*CDH2*)-positive EEC stem cells was identified in the basalis during both proliferative and secretory phases [15]. Active WNT and FGF signaling has been suggested to contribute to an endometrial stem cell niche in the basalis glands [15,32]. Similarly, one EEC progenitor line also expressing *CDH2* (cadherin-2) was identified in NEM and EUE but was absent in EMS lesions [14]. The role of endometrial basalis stem cells in EMS remains unclear, because the basalis is not shed during menstruation. This limitation similarly applies also to endometrial stromal stem cells located in the basalis.

#### 3.7.3. Vascular and Perivascular Stem Cells

The Stro-4 vascular smooth muscle progenitor cells share similarities with ECs and showed the highest abundance in the early secretory phase [16]. The authors proposed two potential sources of the uterine ECs: the bone marrow and the ESCs. Furthermore, they suggested that Endo-3 could be the precursor for two different developmental pathways: the first leads to Endo-4 and subsequently to Endo-6, while the second proceeds via Endo-5 to Endo-7.

Mesenchymal perivascular progenitor cells expressing the Sushi domain-containing protein 2 (*SUSD2*) have been identified in the SMC group [9], NEM, EUE, PE, and adjacent peritoneal tissue, but not in OMA [21]. To our knowledge, the presence of *SUSD2* in endometrial cells in EMS lesions has not yet been demonstrated.

### 3.8. Comparison of Normal (NEM) with Eutopic (EUE) and Ectopic Endometrium (EMS)

The primary objective of this review was to determine whether scRNA-seq can provide an endometrial cell atlas for the normal, eutopic and ectopic endometrium, and whether it is possible to get insights into the pathogenesis of endometriosis. Of note, currently and to the best of our knowledge, no one has attempted to histologically determine the cell proportions of the various endometrial cell types. It must be critically noted that the two endometrial cell atlases [15,16] diverge significantly from one another, both in terms of cell proportions and marker analysis. Despite these shortcomings, it was possible to identify several aspects regarding the pathogenesis of EMS. First, we compared NEM with EUE in terms of the initiation of EMS. In a second step, we compared NEM/EUE with EMS to identify disease-associated alterations.

- Comparison of NEM with EUE
  1. We could establish the following order in terms of cell type frequency: ESCs, EECs, ICs, ECs, and PVs.
  2. The differences between NEM and EUE are described differently and range from few [15,19] to many [17,20,21].
  3. Proportions of ESCs and EECs were only moderately different in EUE vs. NEM.
  4. The proportion of ECs in EUE was significantly reduced, which might result in a thinner endometrium and thus contribute to infertility in women with EMS.
  5. Particularly striking was the significantly increased proportion of ICs in EUE compared to NEM, presumably caused by macrophages/monocytes.
  6. The identification of ESCs, EECs, ECs and PVs with stem cell characteristics still needs confirmation that these cells are indeed stem cells.
  7. Interestingly, no nerve cells were detected in NEM or EUE.
  8. NEM clustered with ~50% EUE, whereas the remaining EUE clustered with OMA [22].
  9. Decidual cells were significantly more abundant in EUE than in NEM [15].
  10. An increased expression of (pro-)inflammatory genes in uM1 was found in EUE [15].
  11. Pathogenic changes in ESCs play a key role, indicating that abnormalities in EUE contribute to the initiation of ectopic EMS lesions [17].
  12. EUE is a transition state supporting the implantation theory [19].
  13. The EUE tissue was divided into two distinct groups differing in the frequency of ICs or Fibs [21].
  14. A dysregulation of interaction of ESCs with ICs in the EUE compared to NEM was reported [15,17].

Summary: Most authors agree that NEM and EUE are more similar than NEM/EUE with EMS. An increased number of decidual cells and ICs was found in EUE vs. NEM, and communication between ESCs and ICs appears to be disrupted in EUE.

B. Comparison of NEM/EUE with EMS
  1. The EEC count in particular was dramatically reduced in EMS.
  2. The proportion of ECs was significantly increased in EMS in contrast to PVs.
  3. No nerve cells were mentioned in EMS, except Schwann cells in DIE.
  4. Most studies reported substantially greater differences between NEM and EMS than between NEM and EUE [17,18], except for one study [21].
  5. Two subtypes of decidualized stromal cells and macrophages were identified as EMS-relevant [15].
  6. EMS-specific MyoFib and Fib subpopulations were found in EMS [18]. The MyoFibs were enriched in TGF-beta receptor signaling and wound healing pathways [19].
  7. EMS lesions showed a significantly altered cellular composition and gene expression, with few EECs and a dominance of ESCs [21].
  8. In OMA, several genes in ESCs, including ECM reorganization and multiple collagens, were upregulated [22].
  9. The overall cellular composition of EMS was not significantly associated with DIE, PE, fibrosis or hemorrhage [22].
  10. Both EECs and ESCs exhibited marked differential gene expression associated with DIE or PE status [22].
  11. The proportion of ciliated EECs in EMS/OMA is increased [20].
  12. An increased proportion of ECs in EMS might result in a highly vascularized microenvironment, along with evidence of vascular dysregulation [21].
  13. ECs in DIE showed angiogenesis, pro-inflammation, and signatures for leaky permeability [18].
  14. A PV cell type specific to PE lesions might play a dual role in promoting angiogenesis and transporting ICs [21].
  15. A higher expression of ERbeta and a decreased PGR expression or diminished progesterone signaling signatures in EMS lesions point to a dysregulated hormonal environment [17,18].
  16. The distribution of proliferative and secretory ESCs was not associated with the menstrual cycle phases, mirroring the dysregulated relationship with hormones [22].
  17. The innate immune system was profoundly dysregulated in EMS [18,21].
  18. Cells in DIE lesions showed profoundly altered inflammatory signatures [18].
  19. The proliferative activity in EMS was very low [19].
  20. The abundance of nicotinamide N-methyltransferase (*NNMT*) in EECs in EUE and EMS is higher compared to NEM [20].
  21. EMS cells reshape the local microenvironment extensively [22].
  22. Comparison of OMA with PE or OMA with PE and with DIE showed many differences by gene expression [18,21,22] or by subpopulations of macrophages, MyoFibs/Fibs, and ECs [18].
  23. The percentages of tissue-resident macrophages rose dramatically—doubling from NEM to EUE and increasing 10-fold in OMA compared to NEM [21].

Two critical remarks: Point 19—Another study found that in EMS the proliferative activity is increased [33]. Point 20—Hou et al. [34] detected *NNMT* mainly in endometrial ESCs and not in EECs.

Summary: Compared with NEM/EUE, EMS lesions showed a different cellular composition, characterized by a shift from Fibs to MyoFibs, suggesting fibrosis. Additionally, angiogenesis (e.g., leaky permeability), inflammation, the hormonal environment and the immune system were dysregulated in EMS.

Before drawing the final conclusions, it is important to highlight and evaluate individual aspects. Until now, it was only known that EMS is primarily associated with dysregulation of decidualization [35], whereas an increased number of decidual cells in the EUE has not been previously reported. This is an important aspect that should be investigated more closely. Another striking finding was the reduction in ECs and PVs in EUE compared to NEM, as it was previously only known that impaired angiogenesis contributes to a thin endometrium in infertile women, but this had not been reported in the endometrium of women with EMS [36].

Brosens et al. [37] reviewed the changes in the endometrium in EUE, which was significantly thinner compared to NEM, but comparable to that of women with recurrent pregnancy losses. This could indicate a defect in the proliferative phase in EUE [38].

The significant increase in the proportion of ICs, especially of tissue-resident macrophages, in EUE compared to NEM is a very interesting observation for the pathogenesis of EMS. In contrast, *APOE*-positive M3 macrophages decreased from NEM to EUE and EMS. This observation is noteworthy, as a lipid-associated, *APOE*-positive macrophage subtype has been identified and in experiments with *APOE* gain-of-function in mice, these cells reduced the size of endometriotic lesions as well as fibrosis [39]. These shifts suggest that the primary cause is more likely to be a dysregulation of the immune system than alterations in the cell proportions of ESCs and EECs. It should be noted that the macrophage subtypes described in various studies can only be compared with caution. Depending on signals from the microenvironment, macrophages exhibit high plasticity, characterized by a polarization continuum between M1 and M2 states [40]. Macrophages at the M1 end of the spectrum possess a pro-inflammatory phenotype with pronounced phagocytic and cytotoxic activity. In contrast, M2 macrophages are primarily involved in the resolution of inflammation and tissue repair. Compared to M1, M2 macrophages are functionally more diverse; various subtypes (M2a–M2d) exist that express distinct cytokines, chemokines, and growth factors. Furthermore, macrophages have a dual origin: they are either tissue-resident or derived from the bone marrow. Tissue-resident macrophages can be identified by the expression of *FOLR2*. However, to date, this marker has been used in only four of the scRNA-seq publications cited in this review [15,18,21,39]. Macrophage polarization and the resulting imbalance between M1 and M2 macrophages significantly influence inflammatory diseases such as endometriosis [41,42].

When comparing NEM/EUE with EMS, the EEC count in particular was dramatically reduced. This result is highly unlikely, as EECs do not disappear as significantly in EMS histologically. The high degree of heterogeneity in the scRNA-seq datasets has already been noted, with the distinct cell isolation procedures identified as a significant factor [15]. Variations in cycle phases and hormonal therapy have also been highlighted as additional causes of the observed differences [15]. We took these factors into account wherever possible; cases involving hormonal therapy were excluded, and cycle phases were considered in our analysis. With the exception of a marked increase in IC numbers, particularly during the proliferative phase in EUE compared to NEM, there appear to be no EMS-specific cell populations, leading to the hypothesis that the differences between NEM and EUE are of a more subtle nature [15].

A notable finding was the marked increase in NK cells, mast cells, and macrophages/monocytes, while the number of T cells and neutrophils was reduced in EMS compared to NEM/EUE. Additionally, alterations in immune-relevant signaling pathways were observed. Endometrial cell alterations have often been suspected as triggers of EMS, but profound changes are rare [3,43]. Endometrial cells change much less than tumor cells [3]; for example, they have very few mutations and there is no epithelial–mesenchymal transition (EMT) in the endometrium that could be responsible for the development of the disease [30]. Furthermore, a recent meta-analysis found only minor differences of 0.92% in gene expression between NEM and EUE [3]. Endometrial cells change their phenotype slightly in the endometrium, as well as their genotype, although there are undoubtedly differences in protein patterns, such as *BCL6* [44] or progesterone resistance [45], to name but a few.

Since none of the authors were able to detect nerve cells or stem cells in EMS, this may be due either to the fact that the currently employed methods lack sufficient sensitivity, or that stem cells play no role in the pathogenesis of endometriosis. We have already pointed out the problems with the stem cell hypothesis for the pathogenesis of endometriosis [46], but a satisfactory answer has yet to be provided.

The two main problems are that the stem cell must differentiate into two distinct cell types, endometrial epithelial and stromal cells, and this must happen in an identical manner at many different sites in the body. Furthermore, no transformation of stem cells into endometrial epithelial and stromal cells has ever been shown to occur at the sites of ectopic endometrial implants.

The fact that no nerve cells were detected in endometriotic lesions, neither in the studies presented here nor in more recent scRNA-seq studies, should not obscure the relevance that they are key components of EMS-associated pain. This encompasses aspects such as neuro-angiogenesis, nerve association with implants, nerve compression by implants, increased nerve fiber density, and neuro-immune interactions [47]. Nevertheless, the failure to detect nerve cells demonstrates that the sensitivity of scRNA-seq is currently insufficient to fully characterize all cell types.

Our analysis of the scRNA-seq data suggests that the primary cause of EMS in the endometrium is more likely a significant increase in IC abundance, particularly macrophages, than a change in the cell proportions or phenotypes of the endometrial cells. This is a fundamental difference between endometrial cells in EMS and tumor cells [3]. Naturally, this hypothesis requires verification in further studies, but there are several supporting observations. The important role of the immune system was demonstrated in a mouse model where the initiation of EMS was immune-mediated but independent of estrogen, while lesion growth was estrogen-dependent. This corresponds to hormonal cycles, as sex steroid hormone concentrations are low and inflammatory activity is high during menstruation [48,49]. The significant increase in the proportion of immune cells at the onset of EMS has not been given sufficient attention, although the role and increase in proportions of M1 or M1/M2 inflammatory profile in the EUE compared to the NEM was reported [50,51]. A recent transcriptome analysis showed a significant downregulation of immune-related signaling pathways in EMS compared to NEM, but also disease-specific immune signatures [52]. Supporting our concept, EMS lesions were significantly reduced by depleting a subtype of macrophages, the Tet methylcytosine dioxygenase 3 (*TET3*)-expressing macrophages [53]. Targeted treatment of such macrophage subpopulations could also be a promising therapeutic approach to prevent the initiation of endometriosis in the endometrium. In our opinion it is important to limit immunotherapy to the early stages of endometriosis, as the first immunotherapy for deep-infiltrating lesions using a TNF-alpha inhibitor reduced neither the lesions nor the pain compared to a placebo [54]. Furthermore, it should be noted that there are least two aspects of endometriosis that may be resistant to immunotherapies, specifically fibrosis [55] and neuro-angiogenesis [56]. Currently there are no therapies for humans for either of these two pathologies.

Comparison of the different EMS entities with EUE and/or NEM revealed significant differences in both cellular proportions, such as macrophages, MyoFibs, ECs [18], and gene signatures [18,21,22]. These differences were not as clearly described when comparing NEM with EUE. This reinforces our conclusions drawn from our analysis of EMT and gene expression in NEM, EUE and EMS [3,57]. The cellular changes as well as the cell proportions of endometrial cells occur primarily after, and clearly less before, implantation into ectopic sites and might be due to the distinct microenvironments.

#### Limitations, Unresolved Questions and Future Research Priorities

The heterogeneity of the cells, cell proportions, and marker analyses limits the conclusions that can be drawn from the review. We therefore question the validity of all conclusions regarding the various pathogenic mechanisms described in the studies examined. A case in point is the discrepancy in how the similarity between NEM and EUE, and between NEM/EUE and EMS, is assessed. Consequently, our analysis can only identify a trend that may be confirmed or refuted by future studies.

The initial cell atlases of the endometrium and endometriosis demonstrate the critical importance of standardizing cell isolation methods and enhancing sensitivity. Standardizing marker analysis is equally important, given the extensive cellular heterogeneity. A particularly notable finding was that isolating EECs from endometriosis samples yielded an insufficient cell count. Identifying the source of error in the isolation process is crucial to enabling more accurate conclusions regarding cell proportions. Our extensive experience in isolating EECs from testicular [58] and endometrial tissue [59] has shown that the isolation process should be as gentle as possible.

## 4. Conclusions

**Figure 1:**
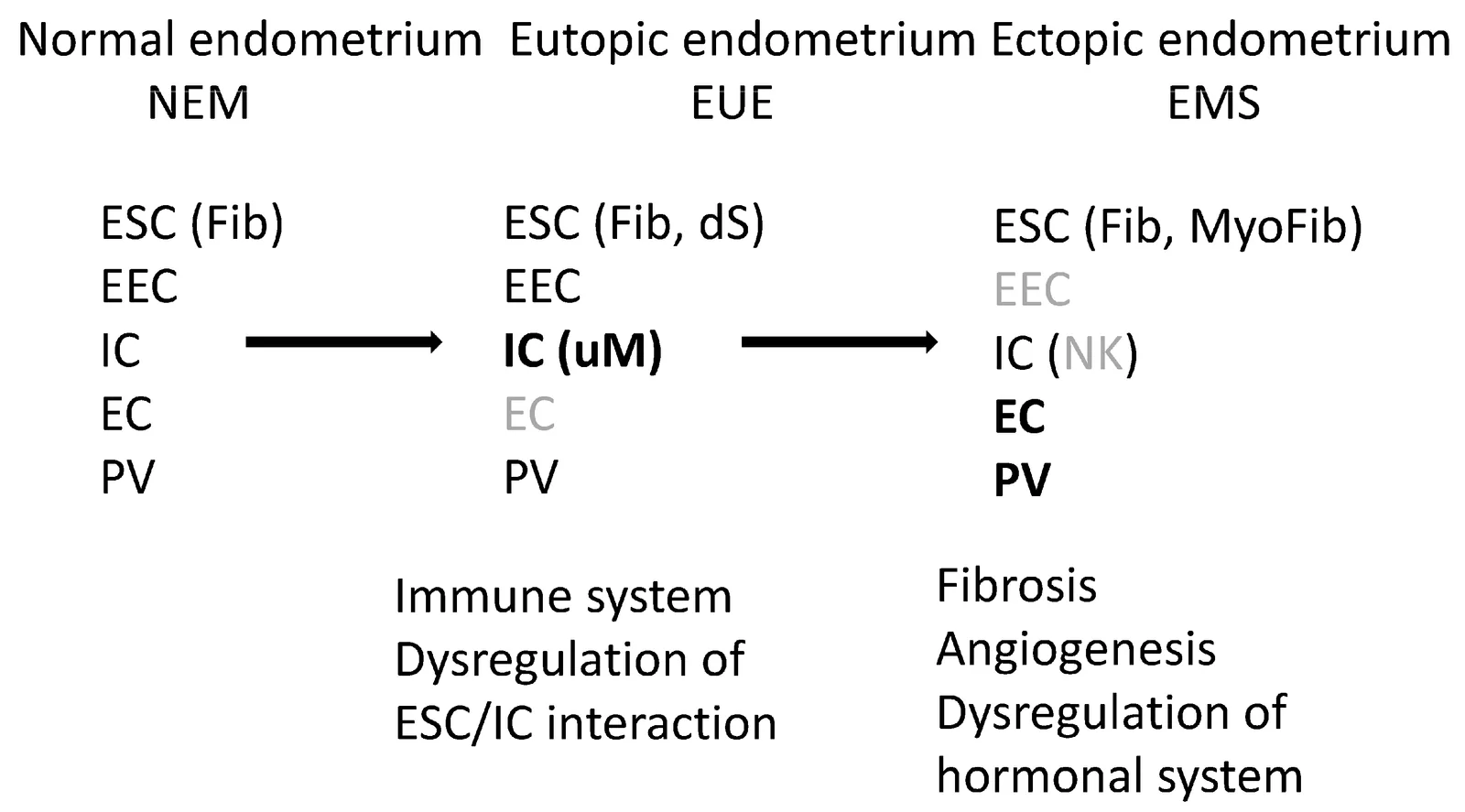
Cell proportions in NEM, EUE, and EMS. For the sake of clarity, we have only emphasized the most important changes in ICs (bold letter) and decidual cell (dS) proportions together with the dysregulation of the ESC/IC interaction in EUE. In the three EMS entities, the EEC proportions (gray letter) are markedly decreased; however, we are skeptical about this strong decrease. The proportions of ECs/PVs and MyoFibs are increased (bold letters) indicating angiogenesis and fibrosis, respectively. Bold letters denote increase and gray letters decrease; NEM, normal endometrium; EUE, eutopic endometrium; EMS, endometriosis; ESCs, endometrial stromal cells; EECs, endometrial epithelial cells; ICs, immune cells; ECs, endothelial cells; PVs, perivascular cells; NKs, natural killer cells.

In summary, the scRNA-seq data show subtle changes in the stromal and epithelial cell proportions, but markedly increased numbers of immune cells and associated changes in the eutopic endometrium (Figure 1). Thus, the data are consistent with Sampson’s implantation hypothesis. Recognizing the significant role of the immune system in the initiation of endometriosis could change our perspective and shift our focus to new treatment options that address the changes in the immune environment at the onset of endometriosis in the eutopic endometrium.

## Figures and Tables

**Table 1:** Characteristics of patients used for scRNA-seq.

Ref	NEM	EUE	OMA	PE	DIE	Age	OC
[9]	7P/12S	n.d.	n.d.	n.d.	n.d.	18–34	no
[10]	3P	n.d.	n.d.	n.d.	n.d.	24–32	no
[11]	3P/1 *	2	n.d.	n.d.	n.d.	n.r.	n.r.
[12]	3P	n.d.	n.d.	n.d.	n.d.	27–31	n.r.
[13]	3S/9if	n.d.	n.d.	n.d.	n.d.	29–35	no
[14]	3P/4S	3P/3S	n.d.	n.d.	n.d.	24–33	no
[15]	5P/1S/1M	2P/2S/5M	n.d.	n.d.	n.d.	22–40	no
[16]	2P/1S/1OC	n.d.	n.d.	n.d.	n.d.	28–31	yes
[17]	3P	3P	3P	n.d.	n.d.	26–34	no
[18]	6P/13S	n.d.	4T	6T	7T	23–49	no
[19]	3S	3S	3S	n.d.	n.d.	29–46	no
[20]	5P/6S ^$^	9P/8S	12P/11S	n.d.	n.d.	26–48	no
[21]	1P/2OC	1M/8OC	4OC	1/7OC/6 ctrl (ad)	n.d.	22–45	yes
[22]	3OC	3P/2S/2OC	4P/2S/2OC	5P/6S/1OC	1P/3S/1OC	24–62	yes

Ref, reference; P, proliferative; S, secretory; M, menstrual; EUE, eutopic endometrium; NEM, normal endometrium; OMA, ovarian endometriosis (endometrioma); PE, peritoneal endometriosis; DIE, deep-infiltrating endometriosis; OC, hormonal contraception; n.d., not done; n.r., not reported; ad, adjacent; ^$^ two patients with teratoma; * unknown cycle; if, infertile; T, tissue samples; ctrl, controls.

**Table 2:** Overview of the identified endometrial cell types.

Ref	ESCs	EECs	ECs	PVs	ICs	Cell No.
[9]	2	2	1	n.r.	2	710,332
[10]	10	1	1	2	2	6348
[11]	1	4	1	2	5	98,568
[12]	2	3	1 + 1 L	1	7	29,373
[13]	7	4	2	1	5	60,222
[14]	9	9	4	n.r.	8	138,036
[15]	7	13	3	3	2	~76,000
[16]	11	9	5 + 1 L	3	10	17,886
[17]	13	1	2	n.r.	12	55,188
[18]	10	9	7	4	27	239,221
[19]	4	2	2	1	9	46,445
[20]	2	4	1	n.r.	3	7030
[21]	8	10	4 + 1 L	3	29	108,497
[22]	13	8	n.r.	n.r.	n.r.	373,851

The numbers denote the numbers of distinct cell types. Ref, reference; ESCs, endometrial stromal cells; EECs, endometrial epithelial cells; PVs, pericytes/perivascular cells; ECs, endothelial cells; ICs, immune cell population; L, lymphatic; n.r., not reported.

**Table 3:** ESC cycle phase-specific relative cell abundance in NEM [16].

	Proliferative	Late Secretory	Changes
Stro-5 (*RUNX3* Fib)	0.6	0.2	Decreased (3×)
Stro-6 (uSMC)	3.2	12.2	Strongly increased (3.8×)
Stro-7 (C7 Fib)	0.2	0.8	Increased (4×)
Stro-8 (dS1)	0.9	7.2	Strongly increased (8×)
Stro-9 (dS2)	33.3	47.7	Increased (1.4×)
Stro-10 (dS3)	46.7	23.7	Decreased (2×)
Stro-11 (stromal progenitor)	6.6	0.3	Strongly decreased (22×)

The numbers denote the relative cell abundance as given by the authors. ESC, endometrial stromal cells; NEM, normal endometrium; dS, decidual stromal; Fib, fibroblast; uSMC, uterine smooth muscle cells; Stro, stromal.

**Table 4:** Phase-specific relative cell abundance of EECs in NEM [16].

	Proliferative	Late Secretory	Changes
Epi-1 ciliated	20.6	22.6	Unchanged
Epi-2 gland_secretory	8.9	15.0	Increased (1.7×)
Epi-3 *SOX9*_glandular	24.4	2.5	Strongly decreased (9.8×)
Epi-4 lumenal	13.2	10.1	Unchanged
Epi-5 gland_sec_progenitor A	11.9	26.1	Increased (2.2×)
Epi-6 gland_sec_progenitor B	3.1	15.1	Strongly increased (4.9×)
Epi-7 myoepithelial	2.6	2.8	Unchanged
Epi-8 *OLFM4*_proliferative	13.6	3.2	Strongly decreased (4.3×)
Epi-9 tissue-res mem T cells	1.8	2.5	Unchanged

The numbers denote the relative cell abundance as given by the authors. EECs, endometrial epithelial cells; NEM, normal endometrium; Epi, epithelial; res mem, resident memory; gland_sec, glandular secretory.

**Table 5:** ECs and PVs in the proliferative and late secretory phase of NEM [16].

	Proliferative	Late Secretory	Changes
Stro-1	2.6	0.9	Decreased (2.9×)
Stro-2	2.0	3.3	Increased (1.7×)
Stro-3	3.5	3.3	Unchanged
Stro-4	0.5	0.4	Unchanged
Endo-1—artery	8.3	4.9	Decreased (1.7×)
Endo-2—EndoMT	14.0	7.3	Decreased (1.9×)
Endo-3—PCV	17.7	16.7	Unchanged
Endo-4—tPCV	7.0	15.1	Increased (2.2×)
Endo-5—capillary	13.5	11.8	Unchanged
Endo-6—aPCV	1.3	26.9	Strongly increased (20.7×)
Endo-7—Tip	14.1	2.5	Strongly decreased (5.6×)
Endo-8—HEV	24.1	14.9	Decreased (1.6×)

The numbers denote the relative cell abundance as given by the authors. NEM, normal endometrium; ECs, endothelial cells; PCV/PVs, perivascular cells; EndoMT, ECs undergoing mesenchymal transition; HEV, high endothelial venule.

**Table 6:** ICs in the proliferative and late secretory phase of NEM [16].

	Proliferative	Late Secretory	Changes
Imm-1: Mast cells	3.6	4.5	Unchanged
Imm-2: Fibrocyte	2.2	1.4	Decreased (1.6×)
Imm-3: TMEMCD8	13.8	7.8	Decreased (1.8×)
Imm-4: TMEMCD4	9.4	8.3	Unchanged
Imm-5: Treg	1.4	0.8	Decreased (1.8×)
Imm-6: ILC	7.0	1.6	Strongly decreased (4.4×)
Imm-7: B cells	0.4	2.0	Increased (5.0×)
Imm-8: Macrophage	17.5	25.2	Increased (1.4×)
Imm-9: NK cells	41.1	43.6	Unchanged
Imm-10: Hematopoietic precursor	3.4	4.8	Increased (1.4×)

The numbers denote the relative cell abundance as given by the authors. ICs, immune cells; NEM, normal endometrium; ILC, innate lymphoid cells.

**Table 7:** Percentages of the endometrial cell types in NEM and EUE [15].

	ESC	EEC	IC	EC	PV
NEM P%	57.80	14.57	12.31	8.43	6.88
EUE P%	47.51	20.93	21.92	5.61	4.02
NEM S%	51.11	28.02	7.33	9.39	4.16
EUE S%	59.60	23.78	10.77	2.79	3.06

NEM, normal endometrium; P, proliferative; S, secretory; EUE, eutopic endometrium; ESC, endometrial stromal cells; EEC, endometrial epithelial cells; IC, immune cells; EC, endothelial cells; PV, perivascular cells. Details are given in Table S3.

**Table 8:** Proportions of subtypes of ESCs in NEM versus EUE [15].

	NEM (%)	EUE (%)	Changes
eStromal MMPs	3.2	2.2	Decreased (1.5×)
eStromal	60.6	40.5	Decreased (1.5×)
eStromal cycling	7.5	6.7	Unchanged
dS-early	6.9	17.4	Increased (2.5×)
dS-mid	20.4	33.0	Increased (1.6×)
dS-late	1.6	0.3	Strongly decreased (5.3×)

ESCs, endometrial stromal cells; NEM, normal endometrium; EUE, eutopic endometrium; dS, decidual stromal; dS-early, dS-mid, dS-late; early secretory phase, mid-secretory phase, late secretory phase, respectively.

**Table 9:** Proportions of subtypes of EECs in NEM compared to EUE [15].

EEC Type	NEM (%)	EUE (%)	Change
SOX9 Basalis	0.06	1.25	Strongly increased (20.8×)
SOX9 Funct I	0.13	0.52	Increased (4×)
SOX9 Funct II	35.8	24.1	Decreased (1.5×)
SOX9 Lumenal	3.74	4.38	Unchanged
Cycling	5.58	12.62	Increased (2.3×)
Pregland	18.2	21.4	Unchanged
Gland	5.6	26.1	Strongly increased (4.7×)
Gland_sec	18.62	1.89	Strongly decreased (9.9×)
Gland_sec, FGF7	0.31	0.13	Decreased (2.4×)

EECs, endometrial epithelial cells; NEM, normal endometrium; EUE, eutopic endometrium; Funct, functionalis; Pregland; pre-glandular; gland, glandular; sec, secretory.

**Table 10:** Percentages of the endometrial cell types in NEM, EUE and EMS.

Ref	ESC	EEC	IC	EC	PV
NEM					
[18]	44.21	34.74	8.95	6.32	6.05
[19]	56.66	12.35	27.6	2.18	1.21
[20]	41.05	22.28	33.77	2.90	n.r.
Mean	47.3	23.1	23.4	3.8	3.6
EUE					
[18]	50.13	1.06	23.48	12.40	9.50
[19]	69.42	11.65	13.11	4.37	1.46
[20]	34.43	18.44	46.47	0.66	n.r.
Mean *	51.3	15.05	27.7	5.8	5.5
EMS					
[18]	52.66	1.15	8.85	11.67	9.69
[19]	40.10	0.29	33.95	11.16	14.5
[20]	48.08	4.24	41.66	6.02	n.r.
Mean	46.95	1.9	28.2	9.6	12.1
Mean **	44.0	15.0	25.3	6.7	9.1

NEM, normal endometrium; EUE, eutopic endometrium; EMS, endometriosis; Ref, reference; ESC, endometrial stromal cells; EEC, endometrial epithelial cells; IC, immune cells; EC, endothelial cells; PV, perivascular cells; * corrected mean, without the outlier; ** corrected means under the assumption that the decrease in EECs from NEM to EMS is 35%.

**Table 11:** Percentages of T cells, NK cells and macrophages in NEM, EUE and EMS.

Ref	NEM	EUE	EMS
T cells (%)			
[17]	45.5	36.5	53.4
[19]	49.7	15.8	41.0
[20]	8.54	15.54	17.6
Mean (%)	34.6	22.6	37.3
NK cells (%)			
[17]	42.3	41.3	10.6
[19]	32.3	44.4	4.7
[20]	15.9	14.5	2.84
Mean (%)	30.2	33.4	6.0
M cells (%)			
[17]	5.8	8.5	23.3
[19]	19.7	39.7	54.3
[20]	9.35	16.45	21.26
Mean	11.6	21.6	33.0

NEM, normal endometrium; EUE, eutopic endometrium; NK, natural killer cells; EMS, endometriosis; Ref, references; M, macrophages/monocytes.

**Table 12:** Percentages of ECs in NEM and EMS [18].

	NEM	OMA	PE	DIE	Changes
Venous-1	9.6	23.5	24.7	35.5	Strongly increased
Venous-2	36.6	10.1	15.2	10.14	Strongly decreased
Cycling	9.1	1.96	2.8	1.13	Strongly decreased
LEC	1.1	10.1	7.3	3.4	Strongly increased
Arterioles	8.2	13.5	9.0	8.5	Increased only in OMA
Tip	0.55	6.7	5.1	1.5	Increased only in OMA, PE
Capillary	34.7	34.2	36.0	40.0	Increased only in DIE

NEM, normal endometrium; EMS, endometriosis; ECs, endothelial cells; OMA, ovarian endometriosis; PE, peritoneal endometriosis; DIE, deep-infiltrating endometriosis; LEC, lymphatic endothelial cells.

## Data Availability

No new data were created or analyzed in this study. Data sharing is not applicable to this article.

## Supplementary Materials

The following supporting information can be downloaded at https://www.mdpi.com/article/10.3390/ijms27167440/s1.
