# Supplementary material for: Single-Cell RNA Sequencing Shows Markedly Elevated Immune Cell Proportions in the Endometrium of Women with Endometriosis—A Narrative Review
Source: Int J Mol Sci. 2026 Aug 20;27(16):7440. doi: 10.3390/ijms27167440 (PMC13512989; doi:10.3390/ijms27167440)
Supplement: Supplementary file 1 [file ijms-27-07440-s001.zip › FigS1.pdf]

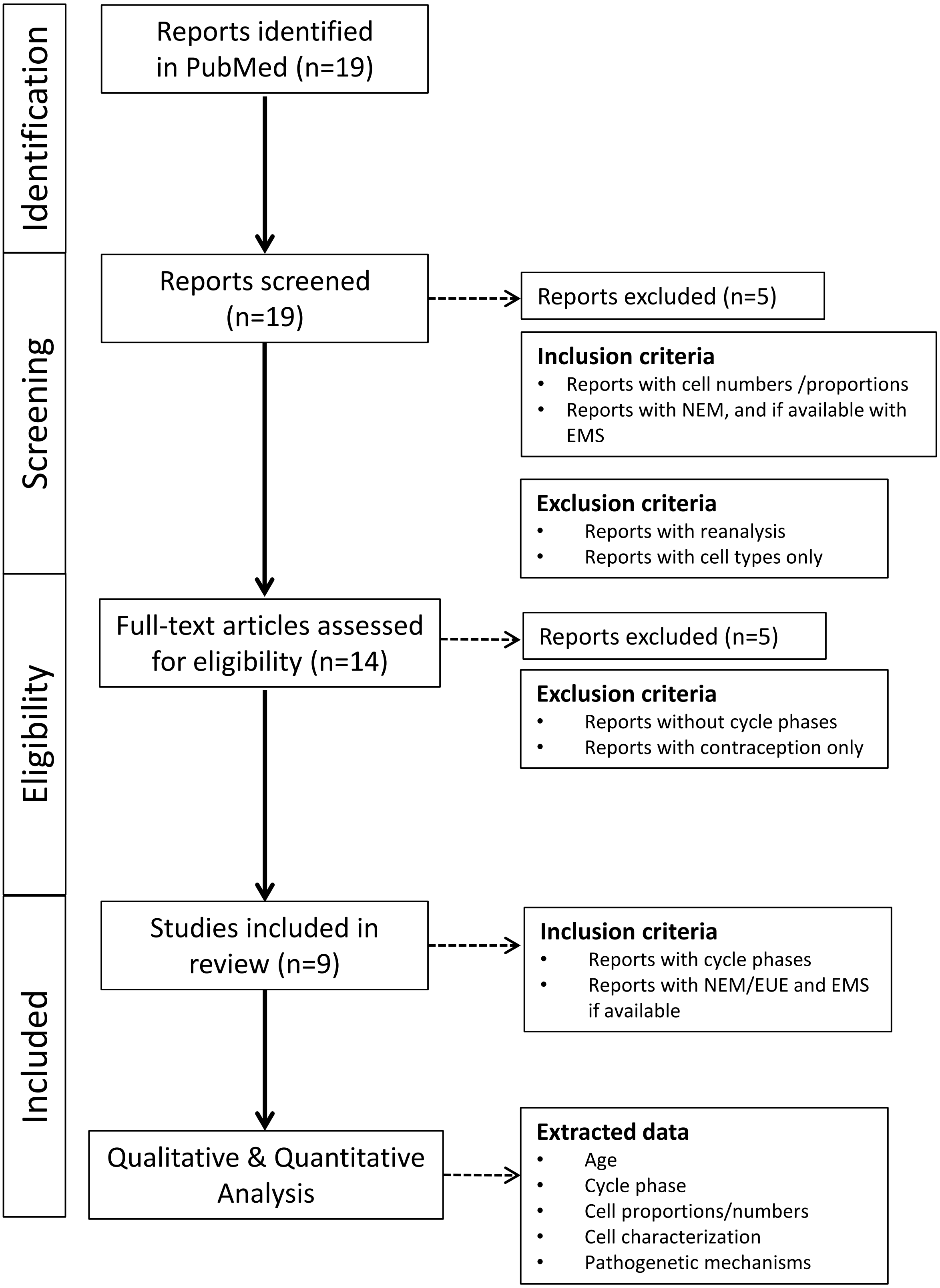

Figure S1. PRISMA flowchart of literature search and data selection. This systematic retrospective review is based upon liter-ature research conducted in PubMed. The main focus was on scRNA-seq in endometrium and if available in EMS with a special focus on cell numbers/proportions. These reports were carefully read and data extracted
